# Residence Time vs. Adjustment Time of Carbon Dioxide in the Atmosphere

**DOI:** 10.3390/e25020384

**Published:** 2023-02-20

**Authors:** Peter Stallinga

**Affiliations:** 1 DEEI, Faculty of Science and Technology, University of the Algarve, 8005-139 Faro, Portugal; peter.stallinga@gmail.com; 2 Ossónoba Philosophical Society, 8000-000 Faro, Portugal

**Keywords:** atmosphere kinetics, residence time, adjustment time, anthropogenic carbon dioxide

## Abstract

We study the concepts of residence time vs. adjustment time time for carbon dioxide in the atmosphere. The system is analyzed with a two-box first-order model. Using this model, we reach three important conclusions: (1) The adjustment time is never larger than the residence time and can, thus, not be longer than about 5 years. (2) The idea of the atmosphere being stable at 280 ppm in pre-industrial times is untenable. (3) Nearly 90% of all anthropogenic carbon dioxide has already been removed from the atmosphere.

## 1. Introduction

One of the major points in discussion of the anthropogenic global warming (AGW) scenario is the time the added carbon dioxide (CO${}_{2}$) stays in the atmosphere. In an extensive study, Solomon concluded that the residence time of carbon atoms in the atmosphere is of the order of 10 years [1], see Table 1. Such a short time would undermine the prime tenet of AGW, since a molecule of CO${}_{2}$ will not have time to contribute to any greenhouse effect before it disappears to sinks where it cannot do any thermal harm. Just as water, a molecule that has orders of magnitude larger greenhouse potency, is irrelevant in the AGW discussion, because any water produced by (non-carbon-only) fossil fuels will rapidly equilibrate and the effect is zero. At best, it will raise ocean levels by some micrometers. As such, if the residence time is below 30 years (the climate window), injections of CO${}_{2}$ in the atmosphere would, just like water, not affect the climate. Or as the IPCC writes in their upcoming report about another atmospheric constituent, “[Water], because of its residence time in the atmosphere averages just 8–10 day, its atmospheric concentration is largely governed by temperature”, the value of 8–10 day coming from Ent [2].

However, some claim that the residence time (the amount of time a molecule on average spends in the atmosphere before it disappears from it) is not relevant for this discussion; what matters is the adjustment time (or relaxation time or (re)-equilibration time), the time it takes for a new equilibrium to establish, the time constant seen in the observed transient, and, allegedly, these two are different. In a recent work, Cawley explains it as [3]

… natural fluxes into and out of the atmosphere are closely balanced and, hence, comparatively small anthropogenic fluxes can have a substantial effect on atmospheric concentrations.

Before we continue and address these points, initially, we need to provide the definitions. According to the IPCC (p. 1457 of Ref. [4]):

Turnover time … is the ratio of the mass of a reservoir (e.g., a gaseous compound in the atmosphere) and the total rate of removal from the reservoir. Adjustment time or response time is the time scale characterising the decay of an instantaneous pulse input into the reservoir. The term adjustment time is also used to characterise the adjustment of the mass of a reservoir following a step change in the source strength. Half-life or decay constant is used to quantify a first-order exponential decay process.

In the current work, we use these exact two concepts, with turnover time called residence time. We also focus on first-order systems mentioned here by the IPCC. We discuss the difference between residence time on the one hand, and adjustment time on the other hand, and test the hypothesis that the adjustment time can be longer than the residence time by mathematical methods. After having addressed this core point, we perform a calculation based on the available data to see how they fit.

## 2. Residence Time and Adjustment Time (Methods, Data, Results and Analysis)

**Figure 1:**
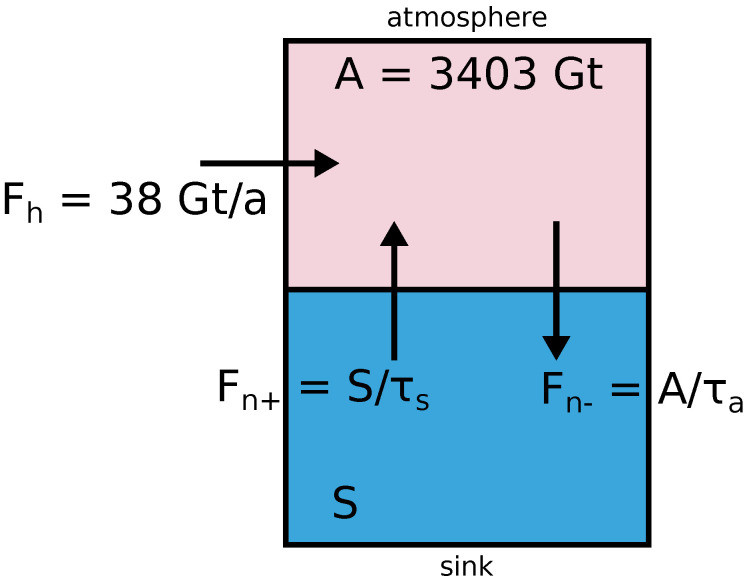
Two-box model of the carbon dioxide cycle. The top box represents the atmosphere, with a total carbon dioxide mass of 3403 Gt. Humans add 38 Gt per year to the system. Nature adds $F_{n+}$ and takes away $F_{n-}$ to a sink represented by the bottom box. That sink has a total CO${}_{2}$ mass equal to *S*. The residence time in the atmosphere, $\tau_{a}$ is well known and estimated to be 5 years, the residence time in the sink $\tau_{s}$ is not well known.

In what follows, we will use a simple two-box first-order model, see Figure 1. The atmosphere has a mass of carbon dioxide equal to *A*. CO${}_{2}$ molecules can be captured into a sink and this occurs at a certain rate, a fraction of the molecules being trapped per time unit. Each individual molecule has a certain probability to be captured over time. In other words, a molecule has a residence time $\tau_{a}$ in the atmosphere (also sometimes called the ’turnover time’), which is the reciprocal of the rate, $k_{a}$. Likewise, in the sink, there is a carbon dioxide mass equal to *S*, where molecules have a residence time $\tau_{s}$; an individual molecule has a certain probability over time to be released by the sink into the atmosphere, or a rate $k_{s}$. This then defines natural fluxes going out of the atmosphere into the sink and vice versa, in a first-order model given by, respectively

(1) $$\begin{array}{ccc} F_{n-} & = & \frac{A}{\tau_{a}},\\ F_{n+} & = & \frac{S}{\tau_{s}}. \end{array}$$

Or in chemistry notation,

(2) $$A\overset{k_{a}}{\underset{k_{s}}{⇌}}S,$$

with $k_{s}=1/\tau_{s}$ and $k_{a}=1/\tau_{a}$. Note that these two time constants are considered constant, independent of time and temperature. At equilibrium, the two fluxes are equal and this links the equilibrium masses to the residence times,

(3) $$\frac{A}{S}=\frac{\tau_{a}}{\tau_{s}}\left(=\frac{k_{s}}{k_{a}}\right).$$

Or, to put it in thermodynamic terms, at equilibrium the change in the Gibbs free energy *G* is zero [5],

(4) $$\Delta G=RT\ln\left(\frac{A}{S}\frac{k_{a}}{k_{s}}\right)=0,$$

with *T* temperature and *R* the gas constant.

Humans add an extra flux into the atmosphere labeled $F_{h}$. On the basis of this, we can determine the adjustment time τ of the atmosphere in terms of the residence times. This requires solving a simple mathematical differential equation; we do not have to worry at this moment about the thermodynamics and explain why the reaction constants are what they are. The questions we ask are, if we add an amount of carbon dioxide ΔA to it:

1. What are the new equilibrium values of *A* and *S*?
2. How long does it take to establish this new equilibrium?

The first question is readily answered. According to Equation (3), the new equilibrium mass in the atmosphere at t=∞ is given by

(5) $$A_{\infty }^{\prime }=A_{0}+\frac{\tau_{a}}{\tau_{a}+\tau_{s}}\Delta A,$$

where the mass before the injection is indicated by the subscript ‘0’, e.g., $A_{0}$. A similar equation can be found for the new amount in the sink (swapping $\tau_{a}$ and $\tau_{s}$ in the expression). For the adjustment time, or relaxation time τ, we use the differential equation

(6) $$\frac{dA(t)}{dt}=F_{n+}-F_{n-}=\frac{S(t)}{\tau_{s}}-\frac{A(t)}{\tau_{a}}.$$

At equilibrium, this derivative is zero and the masses obey the ratio found in Equation (3). If we use the fact that the sum of masses after injection is the sum of masses before injection plus ΔA, and after the injection at t=0 the mass stays constant, then, for t>0

(7) $$S\left(t\right)=\left[A_{0}+S_{0}+\Delta A\right]-A\left(t\right).$$

Substituting this in the equation before, results in

(8) $$\frac{dA(t)}{dt}=-\left(\frac{1}{\tau_{a}}+\frac{1}{\tau_{s}}\right)\left[A\left(t\right)-A_{\infty }^{\prime }\right].$$

The solution of this differential equation is an exponential decay with the new equilibrium value $A_{\infty }^{\prime }$ given by Equation (5), and an adjustment time τ that is the *parallel sum* of residence times, rather than the residence time of only the atmosphere

(9) $$\frac{1}{\tau }=\frac{1}{\tau_{a}}+\frac{1}{\tau_{s}}.$$

As we know from the analogue of parallel electronic resistors, the dominant time constant in this case is the smallest one, and the resulting time constant shorter than the shortest residence time. In other words, the adjustment time of the atmosphere is *shorter* than the residence time of carbon in the atmosphere. To give an example, if the residence time in the atmosphere is 10 years, and the residence time in the sink is 100 years, the adjustment time is 9.1 years. The statement is also true for non-first-order kinetics; no transient can be slowed down by adding a reflux back to the box under study, it would only change the equilibrium value while decreasing the time to reach that. Note: sometimes, the concept of ’half-life’ is also used. It is clear that the time at which half of the perturbation has disappeared is given by $t_{1/2}=\tau \ln\left(2\right)$. The same reasoning used for τ obviously also applies to $t_{1/2}$, with the found time constants multiplied by about 0.69.

**Figure 2:**
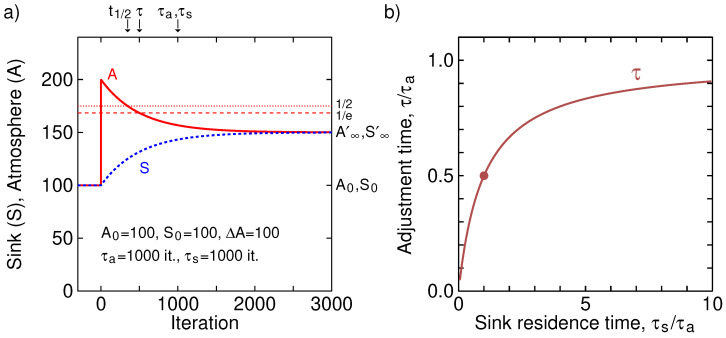
(**a**) A two-box simulation of atmosphere (A) and sink (S) of Figure 1. Before injection of 100 into the atmosphere, the atmosphere-sink system was in equilibrium at 100 each, with the residence ‘times’ in both atmosphere and sink 1000 iterations. At each iteration $A/\tau_{a}$ is moved from atmosphere to sink and $S/\tau_{s}$ moved from sink to atmosphere. As can be seen, the observed adjustment time (relaxation time) of the system is 500 iterations, as predicted by Equation (9). After 500 iterations, the surplus quantity in the atmosphere relative to the new equilibrium has been reduced to 1/*e*, a level indicated by a horizontal dashed line. Further, a half-life can be defined, a time at which half of the transient amplitude has passed, $t_{1/2}=\tau \ln\left(2\right)$ = 347. This is indicated by a dotted line. (**b**) The adjustment time τ, as a function of the sink residence time $\tau_{s}$, normalized by the atmospheric residence time $\tau_{a}$. The dot indicates the value of the plot in (a), $\tau_{s}=\tau_{a}$, resulting in $\tau =\tau_{a}/2$. As can be seen, the adjustment time is shorter than the atmospheric residence time for all values of the sink residence time, with, for large $\tau_{s}$, the adjustment time τ approaching the atmospheric residence time $\tau_{a}$.

Figure 2 shows a simulation of such a two-box system. For better visibility, it is a symmetric system of equal atmosphere and sink with equal residence times. Both atmosphere and sink initially have 100.0 units, and before the first iteration 100.0 units are added to the atmosphere. At each iteration, $A/\tau_{a}$ is moved from the atmosphere to the sink and $S/\tau_{s}$ is moved from the sink to the atmosphere. As can be seen, the observed adjustment time is *half* of the individual residence times, and follows Equation (9). The new equilibrium reached (150 in both atmosphere and sink) is governed by the *total* amount in the sink and atmosphere *after* injection (300) and the ratio of kinetic constants (or reciprocal residence times) $k_{a}=1/\tau_{a}$ and $k_{s}=1/\tau_{s}$. Note that the old equilibrium, with equal amounts of 100 in each box, will never be reached, not even after an infinite amount of time. The characteristic time of the added amount to be ’processed’ and to reach the new equilibrium is what is defined as the adjustment time as, according also to the definition of the IPCC given at the beginning of the text, the time it takes for 1/*e* fraction of the surplus amount, the ’disturbance’ relative to the new (not old) equilibrium, to disappear. This adjustment time is not defined as the time it takes for all added amounts to disappear from the atmosphere. Had we used this latter definition, the adjustment time would be infinite for any value of residence time in the atmosphere and this definition would, thus, be rather meaningless.

We, thus, refute the claim of the climate-skeptics-skeptics [6] that

individual carbon dioxide molecules have a short life time of around 5 years in the atmosphere. However, when they leave the atmosphere, they are simply swapping places with carbon dioxide in the ocean. The final amount of extra CO_2_ that remains in the atmosphere stays there on a time scale of centuries.

Their flawed reasoning is that the adjustment time (relaxation time) is the mass perturbation in the atmosphere divided by the flux *balance*,

(10) $$\tau =\frac{\Delta A}{F_{n+}-F_{n-}},$$

and, so goes the reasoning, while fluxes can be great (and the residence time short), the balance is close to zero and the relaxation time can then approach infinity. Anthropogenic carbon would, thus, be able to stay a long time in the atmosphere. The work of Cawley mentioned before reasons along similar lines, albeit in a more obfuscated way. Equation (5) of that work is similar to the above equation with $F_{n+}$ actually constant. It assumes a non-linear (non-first-order) function of *A* (oddly called a ”linear function” in the work; their Equation (3)) for the outflux $F_{n-}$, a function that is not justified and, moreover, does not make sense; it would imply a non-zero outflux $F_{n-}$ of a system with zero mass *A*. Moreover, the same equation uses the outflux rate (their $k_{e}$, reciprocal residence time) later as the reciprocal adjustment time. They mixed everything up. However, it leads to a relaxation time that can take on any value and could conveniently support a century-scale adjustment time in the presence of a sub-decade residence time, something that physically does not make sense.

In fact, as shown here, the reality is that, if molecules have a residence time in the atmosphere of 5 years, surplus CO${}_{2}$ remains in the atmosphere *less* than 5 years, albeit not much less if the residence time in the sink is much longer. Since that seems to be the case, for all purposes, we can take the residence time as the adjustment time. In fact, we suspect the residence times of Table 1 are actually adjustment times τ, since these are the time constants easily found in a transient, and determining the residence times $\tau_{a}$ from the transients requires more knowledge of the system.

Before we continue, we finish this section by mentioning that the mass ratio of the sink and atmosphere in equilibrium can be estimated from the transient if the injection value of ΔA is known as well as the end value $A_{\infty }^{\prime }$. From that, then the residence time in the sink $\tau_{s}$ can also be established. Looking at Figure 2, or Equation (5), we see that, if $A_{\infty }^{\prime }$, $A_{0}$, ΔA and $\tau_{a}$ are known, $\tau_{s}$ can be estimated, and then the equilibrium ratio is S/A.

## 3. Scenarios

We can now do a more detailed analysis based on the available data. (Note: For easy reading, the pre-industrial values are marked by an asterisk, as in $F_{n+}^{*}$, etc.). We start off with some facts. The pressure at the bottom of the atmosphere is 1020 mbar or hPa ($1.02\times 10^{5}$ N/m${}^{2}$ in S.I. units). This force, divided by the gravitational constant (9.81 m/s${}^{2}$) results in a mass density of $1.04\times 10^{4}$ kg/m${}^{2}$. The total surface area of the planet is 510,072,000 km${}^{2}$; this translates into a total mass of the atmosphere as $5.304\times 10^{18}$ kg. Using a mixture of 20% oxygen (15.999 g/mol) and 80% nitrogen (14.007 g/mol), the average molar mass of air molecules is 28.81 g/mol. The atmosphere, thus, has $1.8408\times 10^{20}$ mol. At this moment, there is a concentration of about [CO${}_{2}$] = 420 ppm (parts-per-million mole fraction) of carbon dioxide in the atmosphere; that is, then, $7.73\times 10^{16}$ mol of CO${}_{2}$. CO${}_{2}$ has a molecular mass of 44.0095 g/mol, so that is a total of $A=3.403\times 10^{15}$ kg. In a similar way, we can say that 1 ppm equals $8.1\times 10^{12}$ kg. A tonne (t) being a thousand kilos, that means 1 ppm is equivalent to 8.1 Gt and there is a total of 3403 Gt in the atmosphere (see Table 2 for factual data on atmospheric carbon dioxide). It also has to be noted that there is sometimes confusion caused by the difference between carbon and carbon dioxide when talking about tonnage. It is clear that a tonne of carbon dioxide is only 273 kg—0.273 tC—of carbon atoms, the rest are oxygen atoms.

**Figure 3:**
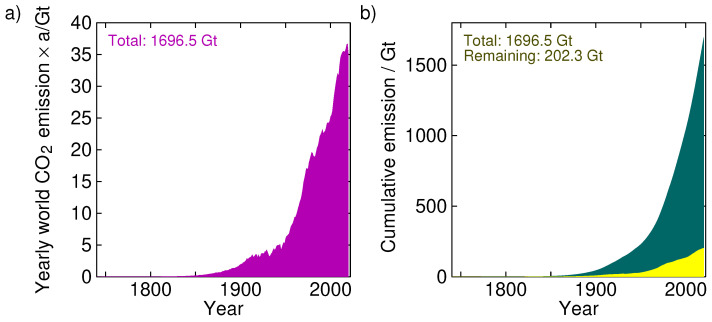
(**a**) Yearly global CO${}_{2}$ emissions from fossil fuels. (**b**) Cumulative emissions (integral of left plot). The yellow curve is the remainder of the anthropogenic CO${}_{2}$ in the atmosphere if we assume a residence time in the sink much longer than the 5-year residence time in the atmosphere; in this case $\tau_{s}=50\tau_{a}$ was used. (Source of data: Our World In Data [8]).

The pre-industrial ’equilibrium’ (axiomatically assuming it indeed was in equilibrium before we started our industry) was 280 ppm. At this moment, every year we inject $F_{h}$ = 38 Gt/a into the atmosphere (see Figure 3a). However, the year-on-year increase in *A* is only about 20 Gt/a [7]. Apparently, about half (47%) immediately disappears, so that there is a net natural flux balance of −18 Gt/a. In our two-box model, the flux goes into the sink without considering the details.

The residence time in the atmosphere can be estimated quite well from the above-ground atomic bomb tests [1], which makes us happy that these at least served the purpose of advancing atmospheric science, if nothing else. The best estimate is about $\tau_{a}$ = 5 years [9]. Other references mention different times, with the IPCC mentioning the shortest (4 years) in their 5th Assessment Report (p. 1457 of Ref. [4]), showing that this value is not settled yet; we will use 5 years in this work. The equilibrium amount of carbon dioxide in the atmosphere is open for debate, but, for this purpose, we might use the consensus value of 280 ppm ($A^{*}$ = 2250 Gt). To estimate the amount of CO${}_{2}$ in the sink is very difficult. However, there seems to be a general view that it is fifty times more than in the atmosphere, S=50A=113,400 Gt (relatively unchanged since pre-industrial times). Using the combination of these values does not allow for consistent bookkeeping, as the reader can easily verify. Something has to yield. In what follows, we will try out some scenarios based on specific assumptions.

### 3.1. Scenario: Pre-Industrial Atmosphere Was at Equilibrium

First we assume that the pre-industrial level of 280 ppm was indeed an equilibrium value with influx equal to outflux in the absence of human flux, as we are wont to believe, but that the mass in the sink *S* and the residence time $\tau_{s}$ in the sink are unknown. Atmospheric carbon dioxide has increased 50% since these pre-industrial times (from 280 to 420 ppm). Since we are dealing with first-order kinetics (Equation (1)), the natural outflux $F_{n-}$ has, thus, also increased 50% from pre-industrial times. The current natural outflux is very well determined at $F_{n-}=A/\tau_{a}$ = 681 Gt/a (both parameters are well-established); in pre-industrial times, it must, thus, have been 33% less, at $F_{n-}^{*}$ = 454 Gt/a. If we maintain the idea that in pre-industrial times the system was at equilibrium, then the natural influx $F_{n+}^{*}$ must have been equal to this outflux $F_{n-}^{*}$ at 454 Gt/a in pre-industrial times and is now found by the flux balance, $F_{n+}$ = 681 Gt/a − 18 Gt/a = 663 Gt/a (46% gain). The residence time of carbon in the sink cannot have changed, so the sink itself must have gained 46% in mass—a conclusion that is highly unlikely since it would imply a rather small carbon buffer in the sink if such tiny flux imbalances can disturb the buffer to such a large extent. In this scenario, the amount of carbon in the sink must be about equal to the amount in the atmosphere.

**Figure 4:**
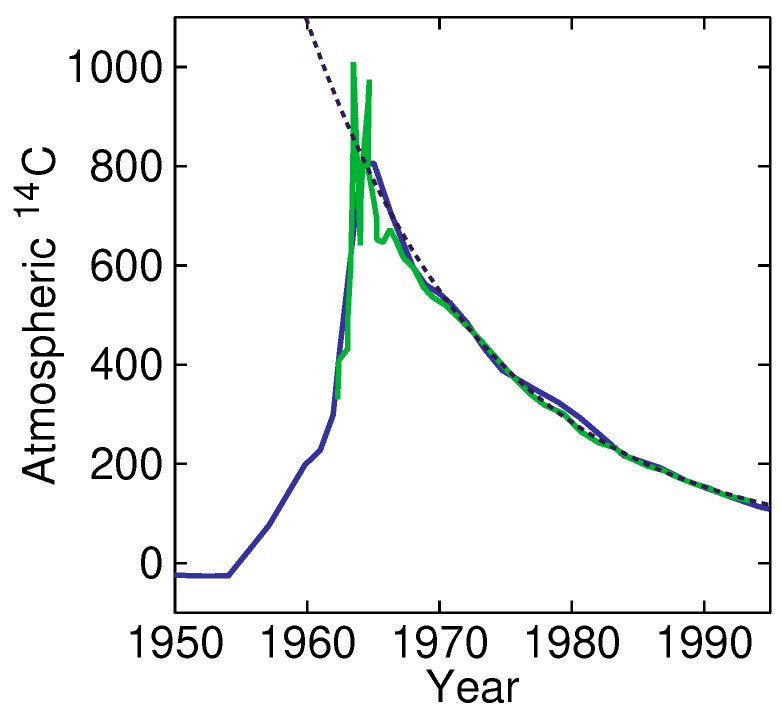
Above-ground atomic-bomb explosions produced a lot of ${}^{14}$C that stopped in the 1960s. From a fit (dashed line) of data from 1965, we find an adjustment time of τ = 14.0 a, and an amplitude of ΔA = 740, with a final value of $A_{\infty }^{\prime }$ = 30. This enables stating that the sink must be at least 24 times larger than the atmosphere. Data from Enting (blue) found in a work of Perruchoud [11] and Nydal et al. [10] (green), extracted with WebPlotDigitizer [12].

Yet, as mentioned before, we can obtain a good estimate from the sink mass in equilibrium from the transients. An especially good tool is the ${}^{14}$C released in the atmosphere by atomic-bomb tests, since this isotope of carbon has very low natural abundance, enabling an accurate estimation of ΔA. Such a partial analysis, with a subset of carbon atoms, is possible, as discussed later. We can actually take the fraction of ${}^{14}$C of all carbon as a measure of the total mass *A* of this subset. Moreover, note that the half-life of nuclear decay of ${}^{14}$C is 5730 years and, thus, no significant decay took place during the experiment; all carbon-14 disappeared from the atmosphere by transfer to the sink. Figure 4 shows an example of investigations carried out by Enting and Nydal [10] (data of Enting from a work by Perruchoud et al. [11]; extracted by WebPlotDigitizer [12]). Using $A_{0}$ equal to zero, from a fit (shown as a dashed line), we find an adjustment time of τ=14.0 a, an amplitude of ΔA = 740, and $A_{\infty }^{\prime }$ = 30. From this, we derive $\tau_{s}$ = 344 a, and $\tau_{a}$ = 14.6 a. We find an equilibrium sink-to-atmosphere mass ratio of 24. This analysis assumes a delta-Dirac insertion of ${}^{14}$C in 1965. From the figure we can see that the ${}^{14}$C-injection already took place earlier and we, thus, underestimate ΔA, and, therefore, underestimate the S/A mass ratio. However, even this lower-estimate can be used to debunk the idea that the sink buffer *S* is small, and, thus, debunk the idea that the atmosphere was stable in pre-industrial times (in this model).

It seems that the idea of the pre-industrial level *stable* at 280 ppm (with $F_{n+}=F_{n-}$ at 280 ppm) is untenable. It seems very likely that the sink was already off-balance and emitting amounts of carbon dioxide at the beginning of the industrial era and the increase in the atmospheric CO${}_{2}$ at any time in human history is not solely due to human activity. This would also explain the large pre-Mauna-Loa values found with chemical methods summarized by Beck [13] and Slocum [14]. For instance, values of 500 ppm have been observed around 1940. Ignoring these facts, on the other hand, would be equivalent to throwing entire generations of scientists under the bus.

### 3.2. Scenario: The Sink Is Fifty Times Larger Than the Atmosphere

Next, we adopt the assumption that the sink at this moment really has 50 times more carbon than the atmosphere, in other words, S=50A = 170,000 Gt, and release the restriction that the atmosphere was stable at 280 ppm; in pre-industrial times there can have been a flux imbalance. We can first make an estimate of the residence time in the sink by noting that the natural outflux $F_{n-}=A/\tau_{a}$ = 681 Gt/a at this moment is not fully compensated by influx from the sink. An imbalance of 18 Gt exists, so $F_{n+}=663$ Gt/a. Given the sink mass, this results in a residence time of $\tau_{s}=S/F_{n+}$ = 256 a. Most of the 1696.5 Gt that we have produced from burning fossil fuels (Figure 3) must have disappeared into the sink. However, that did not make a big dent. In pre-industrial times, the sink mass must have been only 168,000 Gt. The emissions from that sink at that time must have been $F_{n+}=S/\tau_{s}$ = 657.4 Gt/a. The outflux then at 280 ppm ($A^{*}$ = 2250 Gt) was $F_{n-}^{*}=A^{*}/\tau_{a}$ = 450 Gt/a. We see indeed a tremendous *outgassing* from the sink in pre-industrial times. The system was far from equilibrium, with an imbalance being a net influx of $F_{n+}^{*}-F_{n-}^{*}$ = 207 Gt/a. Where, at the moment, there is a net natural flux of 18 Gt/a out of the atmosphere, in pre-industrial times, in this two-box first-order model with a sink 50 times larger than the atmosphere, there was a net natural influx of 207 Gt/a. Somewhere, we must have passed the equilibrium value and, considering the above numbers, this value must be rather close to today’s concentration of 420 ppm.

### 3.3. Scenario: Residence Time in the Sink Is Much Larger Than in the Atmosphere

If we only assume that the residence time in the sink is much larger than in the atmosphere, $\tau_{s}\gg \tau_{a}$, then we can get a good idea of what has happened to our anthropogenic contribution to the carbon in the atmosphere, $F_{h}$, based on the two-box model. Because it is first-order, with all fluxes linearly depending on masses, in our analysis, the carbon dioxide can be decomposed into anthropogenic and natural and each treated separately. In a statistical physics/mathematics analogy, as if one were yellow balls and the other red, and we are constantly randomly taking a fixed fraction (not a fixed number) of balls from one of the boxes and putting them in the other, the chances of getting a yellow or red ball are proportional to the number of balls of that color in the box. A very important observation: adding yellow balls to the system does not change anything about the dynamics of the red balls. Some may think that adding yellow balls in one box (atmosphere) influences the amount of red balls in it, but that is not the case in first-order kinetics; the yellow and red ball subsystems are *fully* independent and can be analyzed separately, even if the observer is colorblind (such that carbon dioxide molecules are indistinguishable). For instance, if the red balls (natural CO${}_{2}$) were at equilibrium before the yellow balls were added, no net flow of red balls will take place after adding them. In other words, we can analyze the anthropogenic and natural CO${}_{2}$ entirely separately and, at the end, simply add them together. The amount of anthropogenic CO${}_{2}$ in the atmosphere does not influence the amount of natural CO${}_{2}$ in the atmosphere and vice versa. We can, thus, analyze how much of the anthropogenic CO${}_{2}$ still remains in the atmosphere by simply analyzing it with our two-box model. (In the case where the atoms can be distinguished, equivalent to being able to see the colors of the balls, we can determine the kinetics parameters by looking at only one type, only one color).

Figure 3 shows the yearly carbon dioxide emissions into the atmosphere (left panel; data source: Our World In Data [8]). The total amount so far emitted is 1696.5 Gt. The right panel shows the cumulative emissions, $\sum_{i}^{\mathrm{year}}F_{h}\left(i\right)$. If at every year we apply the fluxes according to Equation (1), then we can see at each year how much of the anthropogenic CO${}_{2}$ is still in the atmosphere. The right panel of Figure 3 shows this for $\tau_{s}=50\tau_{a}$. We see that only 202.3 Gt of the total injected 1696.5 Gt is still in the atmosphere. In these years, the amount of CO${}_{2}$ in the atmosphere has risen from 280 ppm (2268 Gt) to 420 ppm (3403 Gt), an increment of 1135 Gt. Of these, 202.3 Gt (17.8%) would be attributable to humans and the rest, 932.7 Gt (82.2%), must be from natural sources. In view of this, curbing carbon emissions seems rather fruitless; even if we destroy the fossil-fuel-based economy (and human wealth with it), we would only delay the inevitable natural scenario by a couple of years.

### 3.4. Scenario: Abandoning Constant Residence Times

We have seen here how the first-order-kinetics two-box model results in conclusions contrary to data. We could, of course, change our model. We could abandon the idea of first-order kinetics (where flux is proportional to mass), but that would be problematic to justify with physics. Yet, some authors do that and, in that case, one can add parameters to the system until it has the desired property of having a stable atmosphere at $A^{*}$ = 280 ppm. The chemical measurements described by scientists such as Beck and Slocum mentioned above still remain to be explained. How could we have had very large concentrations in recent history?

We could also add more boxes to the system, distinguishing the sinks, or differentiating between deep ocean and shallow ocean, dissolved carbon dioxide gas, CO${}_{2}$(aq), and dissolved organic carbon (sea-shells), or between CO${}_{2}$ disappearing in the oceans and being sequestered in biological matter on land, etc. Then each box can have its own kinetics; as an example, plant growth is sublinear with CO${}_{2}$ concentration. We leave it for further work to formally analyze the adjustment time in higher-order kinetics systems with any number of boxes.

However, we expect the most likely improvement to the model to come from abandoning the idea that the residence times $\tau_{a}$ and $\tau_{s}$ are constant. They, in fact, are very much dependent on temperature. As an example, the ratio between the two that tells us the concentrations (and, thus, the masses) between carbon dioxide in the atmosphere and in the sink, if we assume this sink to be the oceans, is governed by Henry’s Law, and this concentration ratio is then dependent on temperature. When including such effects, we might even conclude that the entire concentration of carbon dioxide in the atmosphere is fully governed by such environmental parameters and fully independent of human injections into the system. *A* is simply a function of many parameters, including the temperature *T*, but not $F_{h}$. It is as if the relaxation time is extremely short and any disturbances introduced by humans, or by other means, rapidly disappear, rapidly reaching the equilibrium determined by nature.

This fits very nicely with the recent finding that the stalling of the economy and the accompanying severe reduction in carbon emissions during the Covid pandemic had no visible impact on the dynamics of the atmosphere whatsoever [15]. The result of that research, the hypothesis that the carbon dioxide increments in the atmosphere were fully due to natural causes and not humans, fits the experimental data very well, and the hypothesis that humans are fully responsible for the increments can equally be rejected scientifically. This then also agrees with the conclusions of Segalstad that “The rising atmospheric CO${}_{2}$ is the outcome of rising temperature rather than vice versa” [16]. The pre-industrial atmosphere might indeed have been in equilibrium, and we are currently also in, or close to, equilibrium. That seems to us to be the most likely scenario. Once we admit the possibility of non-anthropogenic sources of carbon dioxide, we can start finding out what they might be. Examples such as volcanic sources, planetary and solar cycles spring to mind. It might well be that the climate puzzle is solved in such areas as the link between solar activity and seismic activity and climate [17].

This is, however, not the focus of this work. We conclude here by summarizing the major findings of this analysis using a first-order-kinetics two-box model: (1) The adjustment time is never larger than the residence time and is less than 5 years. (2) The idea of the atmosphere being stable at 280 ppm in pre-industrial times is untenable. (3) Nearly 90% of all anthropogenic carbon dioxide has already been removed from the atmosphere.

## Figures and Tables

**Table 1:** Atmospheric carbon dioxide residence times $\tau_{a}$ presented in [1]. A * indicates author found this value in the referenced publication instead.

Researcher(s)	Residence Time	Researcher(s)	Residence Time
Craig (1957)	7 ± 3 a	Oeschger et al. (1975)	6–9 a
Revelle & Suess (1957)	7 a	Keeling (1979)	7.53 a
Arnold & Anderson (1957)	10–20 a *${}^{1}$	Peng et al. (1979)	5.5–9.4 a
Craig (1958)	7 ± 5 a	Peng et al. (1979)	7.8–13.2 a
Ferguson (1958)	1–8 a	Broecker et al. (1980)	6.2–8.8 a
Bolin & Eriksson (1959)	5 a	Delibrias (1980)	6.0 a
Craig (1963)	5–15 a	Quay & Stuiver (1980)	7.5 a
Bien & Suess (1967)	>10 a	Siegenthaler et al. (1980)	7.5 a
Münnich & Roether (1967)	5.4 a	Stuiver (1980)	6.8 a
Nydal (1968)	5–10 a	Druffel & Suess (1983)	12.5 a
Young & Fairhall (1968)	4–6 a	Kratz et al. (1983)	6.7 a
Rafter & O’Brien (1970)	12 a	Lal & Suess (1983)	3–25 a
Machta (1972)	2 a	Peng et al. (1983)	8.4 a
Bacastow & Keeling (1973)	6.3–7.0 a	Siegenthaler (1983)	7.9–10.6 a
Keeling (1973)	7 a	Siegenthaler (1983)	6.99–7.54 a
Broecker (1974)	9.2 a	Siegenthaler (1989)	4–9 a
Broecker & Peng (1974)	5 a *	Murray (1992)	5.4 a
Broecker & Peng (1974)	8 a	Segalstad (1992)	5.4 a
1: also $\tau_{s}$ = 300 a			

**Table 2:** Carbon dioxide facts, with the natural outflux $F_{n-}$ derived from the mass in the atmosphere and the residence time. Other important parameters, influx $F_{n+}$, sink mass *S*, and sink residence time $\tau_{s}$, are less well known and should be considered adjustable.

Quantity	Parameter	Value
Molecular mass	*m*	44.0095 g/mol
Equivalence	1 ppm [CO${}_{2}$]	8.1 Gt
CO${}_{2}$ mass in atm.	*A*	3403 Gt (at 420 ppm)
Y-o-y change	d*A*/dt	20 Gt/a (in 2020)
Human influx	$F_{h}$	38 Gt/a (in 2020)
Atm. residence time	$\tau_{a}$	5 a
Natural outflux	$F_{n-}$	681 Gt/a (in 2020)

## Abbreviations

The following abbreviations are used in this manuscript:

AGW	Anthropogenic Global Warming
IPCC	Intergovernmental Panel on Climate Change
